# Type 1 regulatory T cell-mediated tolerance in health and disease

**DOI:** 10.3389/fimmu.2022.1032575

**Published:** 2022-10-28

**Authors:** Robert A. Freeborn, Steven Strubbe, Maria Grazia Roncarolo

**Affiliations:** ^1^Division of Hematology, Oncology, Stem Cell Transplantation and Regenerative Medicine, Department of Pediatrics, Stanford School of Medicine, Stanford, CA, United States; ^2^Institute for Stem Cell Biology and Regenerative Medicine (ISCBRM), Stanford School of Medicine, Stanford, CA, United States; ^3^Center for Definitive and Curative Medicine (CDCM), Stanford School of Medicine, Stanford, CA, United States

**Keywords:** Type 1 regulatory T (Tr1) cells, immunological tolerance, autoimmunity, inflammatory bowel disease, infectious disease

## Abstract

Type 1 regulatory T (Tr1) cells, in addition to other regulatory cells, contribute to immunological tolerance to prevent autoimmunity and excessive inflammation. Tr1 cells arise in the periphery upon antigen stimulation in the presence of tolerogenic antigen presenting cells and secrete large amounts of the immunosuppressive cytokine IL-10. The protective role of Tr1 cells in autoimmune diseases and inflammatory bowel disease has been well established, and this led to the exploration of this population as a potential cell therapy. On the other hand, the role of Tr1 cells in infectious disease is not well characterized, thus raising concern that these tolerogenic cells may cause general immune suppression which would prevent pathogen clearance. In this review, we summarize current literature surrounding Tr1-mediated tolerance and its role in health and disease settings including autoimmunity, inflammatory bowel disease, and infectious diseases.

## Introduction

Immunological tolerance is vital in a healthy immune system to prevent excessive inflammation and autoimmunity, and many cell types contribute to tolerance [reviewed in (1)]. Self-tolerance is largely mediated by FOXP3^+^ regulatory T cells (Treg) while type 1 regulatory T cells (Tr1) contribute greatly to peripheral tolerance and to some degree, self-tolerance. Tr1 cells were first discovered when a severe combined immunodeficiency (SCID) patient received a mismatched allogeneic fetal liver and thymus transplant and developed stable mixed chimerism (2, 3). This patient had high levels of serum IL-10, and further analysis revealed that T cell clones from this patient produced substantial amounts of IL-10 rapidly after T cell receptor (TCR) stimulation but produced low amounts of IL-2 (3). Interestingly, IL-2 production was rescued by stimulating the cells in a TCR-independent manner. Follow-up studies established that Tr1 cells were a subset of regulatory T cells that produced large amounts of IL-10 and TGF-β, variable amounts of IFN-γ comparable to undifferentiated CD4^+^ T cells (Th0), and little to no IL-2 and IL-4, therefore making the cytokine profile distinct from Th1 and Th2 cells (4). Tr1 cells proliferated poorly in response to cognate antigen, and this proliferative defect could be rescued by IL-2; it was later demonstrated that Tr1 cells proliferated extensively in response to IL-15 as well (5). Tr1 cells were highly suppressive through secretion of IL-10 and TGF-β, and they suppressed colitis in a mouse T-cell transfer model (4). Secretion of IL-10 can directly limit effector T cell responses, and it can also indirectly suppress T cell responses by limiting the antigen presenting capacity of antigen presenting cells (6–9). We and others showed that Tr1 cells also have cytotoxic potential which can contribute to tolerance *via* elimination of myeloid antigen presenting cells (APC) (10–13). Tr1 cells also suppress through PD-1/PD-L1- and CTLA-4-mediated mechanisms (14). In addition to modulating T cell responses directly and through APCs, Tr1 cells can also regulate antibody production by B cells (15, 16). Due to their potent, antigen-specific suppression, Tr1 cells have garnered considerable attention for clinical applications [reviewed in (17, 18)]. In contrast to FOXP3^+^ Tregs, antigen-specific Tr1 cells can easily be generated *in vitro* and *in vivo*, and polyclonal Tr1 cells can also mediate tolerance *via* modulation of APC function and subsequent induction of antigen-specific Tr1 cells – a process known as infectious tolerance (19). Tr1 cells have clinical applications in inflammatory diseases such as inflammatory bowel diseases (IBD), autoimmune disease (AID) ranging from type 1 diabetes (T1D) to multiple sclerosis (MS), and even in infectious diseases. In this review, we will provide a summary of the role of Tr1-mediated tolerance in health and various disease settings.

## Classification of Tr1 cells and mechanisms of their induction

Tr1 cells are a subset of peripherally induced FOXP3^-^ regulatory CD4^+^ T cells which can limit immune responses to foreign and autoantigens. Prior to the discovery of surface markers to identify Tr1 cells, they were often described based on their hallmark cytokine profile. FOXP3^-^IL-10^+^ CD4^+^ T cells were often referred to as Tr1 cells, though this profile alone is insufficient to identify Tr1 cells. For instance, suppressive IL-10^+^CTLA-4^+^ Th2 cells can arise in the mouse, but these are distinct from Tr1 cells as they secrete high amounts of IL-4, IL-5, and IL-13 (20). In many instances, co-production of IL-10 and IFN-γ has been used to identify Tr1 cells, Tr1-like cells, or Th1 cells expressing IL-10, thus making it difficult to determine the actual cell type being studied. Using co-production of IL-10 and IFN-γ as a metric is not an ideal method to identify Tr1 cells, as human Th1 cells may co-produce these cytokines yet are distinct from Tr1 cells, despite their important role in limiting inflammatory Th1 responses [reviewed in (21)]. It is important to note these differences, as the pathways driving IL-10 production by Th1 cells are impaired in several diseases (22–24). Moreover, if the regulatory capacity of these cells is transient, as has been demonstrated (23), then these cells could potentially reacquire an effector profile and therefore not contribute to resolution of inflammation and could even exacerbate immunopathology. One notable difference between these cell types can be seen by the transcription factors they express: IL-10-producing Th1 cells express high levels of *Tbx21* and *Egr2* but low *Gata3* (25), whereas Tr1 cells isolated from the peripheral blood of healthy donors had elevated *GATA3* expression compared to memory CD4^+^ T cells with no changes in *TBX21* or *EGR2* (26). While high *GATA3* expression was not reported in *in vitro*-differentiated Tr1 cells (27), *TBX21* and *EGR2* expression was not elevated, thus suggesting these may not be features of human Tr1 cells. Additionally, IL-10-producing Th1 cells are often not anergic and proliferate extensively in response to antigen [reviewed in (21)]. Currently, it is unclear how else these two populations may vary. This is an area of research which is being actively pursued utilizing advances in molecular technology including ATAC sequencing to build a better understanding of these cell types.

To better characterize Tr1 cells, extensive efforts aimed to identify cell surface markers unique to Tr1 cells. The first step toward this demonstrated that human CD45RA^-^CD25^-^CD127^-^ CD4^+^ T cells produced IL-10 and IFN-γ upon *in vitro* stimulation, and that these cells were suppressive (28). Interestingly, these cells appeared to have recently undergone activation *in vivo*, as they displayed high expression of Ki67, ICOS, HLA-DR, and CTLA-4. Despite their Ki67 expression, these cells were not proliferative; as with classically described Tr1 cells, the proliferative defect was rescued with IL-2 treatment. Near the same time, another group demonstrated that LAG-3 expression identified a population of murine CD25^-^ CD4^+^ T cells which produced IL-10 and low amounts of IL-2, IL-4, IL-5, IFN-γ, and TGF-β (29). Like Tr1 cells, the CD25^-^LAG-3^+^ CD4^+^ T cells were anergic and capable of suppressing colitis. This phenotype was further refined once transcriptomic analyses were performed on Tr1 clones, revealing that CD49b and LAG-3 were uniquely co-expressed by Tr1 cells (27). Sorting of memory CD49b^+^LAG-3^+^ CD4^+^ T cells from both mice and humans revealed that these were the major IL-10-producing subset of CD4^+^ T cells, and they were highly suppressive *in vitro* compared to other subsets, including CD49b^-^LAG-3^+^ cells, suggesting that these two markers accurately identify Tr1 cells when used together. CD49b^+^LAG-3^+^ CD4^+^ T cells were also enriched in the blood of thalassemic patients who developed persistent mixed chimerism after allogeneic stem cell transplantation (27). Intestinal Tr1 cells from mice and humans also co-express CCR5 and PD-1 (30), and murine CCR5^+^PD-1^+^ cells are nearly all CD49b^+^LAG-3^+^, suggesting these markers work equally well and can likely be used together to more precisely identify endogenous Tr1 cells. Other markers can also aid in the identification of Tr1 cells. For instance, CD226 is highly expressed on Tr1 cells and is important for Tr1-mediated suppression (12). Staining for other co-inhibitory receptors such as Tim-3, TIGIT, and CTLA-4 can also aid in identifying Tr1 cells (31). Beyond being markers for Tr1 cells, LAG-3 and TIM-3 both have minor roles in Tr1-mediated suppression (31). This is consistent with FOXP3^+^ Tregs which have increased suppressive capacity when expressing LAG-3 or TIM-3 (32, 33). Discovery of these markers has permitted rapid progress in the field, but challenges remain. Indeed, many of the markers used to identify Tr1 cells can be induced transiently in cells which adopt a suppressive state (23). Despite this progress, a uniform definition of Tr1 cells still eludes the field, in large part due to the lack of a described lineage-defining transcription factor. Several transcription factors such as cMaf and Blimp-1 were shown to be essential for murine Tr1 differentiation and function (34, 35), but only a couple of these have findings (such as AHR/cMaf regulation of IL-10 production (36)) have been recapitulated in human Tr1 cells. AHR and IRF4 contribute to the suppressive capacity of Tr1 cells (37), but the involvement of these transcription factors in human Tr1 differentiation remains unknown. Eomes is the only transcription factor that has been demonstrated to play a role in the differentiation of a subset of human Tr1 cells (11, 38, 39). However, bulk RNA sequencing revealed that Eomes was not differentially expressed in circulating Tr1 cells compared to non-Tr1 memory CD4^+^ T cells (26), so it is unlikely to be a lineage-defining transcription factor for all Tr1 cells, and instead may only identify a subset of Tr1 cells. The discovery of a universal lineage-defining transcription factor will be a huge leap forward for the field. However, even lineage-defining transcription factors can be insufficient to identify a population. For instance, using FOXP3 to identify Tregs is unreliable, as human effector CD4^+^ T cells upregulate both CD25 and FOXP3 following stimulation; discovery of an epigenetic marker for FOXP3^+^ Tregs overcame this problem (40). Recent studies of Tr1 cells in a mouse model have begun exploring the epigenetic profile of murine Tr1 cells (41), but a unique epigenetic marker has not yet been identified.

Since Tr1 cells are induced in the periphery (29) and are therefore antigen-experienced, they are thought to be memory CD4^+^ T cells and behave as such. Evidence to the contrary was introduced in an airway allergy model in which Tr1-like cells substantially reduced airway inflammation in an acute inflammatory setting but showed no role during re-challenge despite being present as resident memory cells (42). The conclusion that Tr1-like cells did not contribute to tolerogenic memory was primarily based on the finding that inflammation was not enhanced in the recall response upon the *in vivo* depletion of IL-10-producing T cells. However, there were several caveats for this finding: first, the recall response was assessed only one day after challenge and a protective role of Tr1-like cells may not be visible or necessary that early in a recall response; second, the difference in frequency of Tr1-like cells between control or depleted animals was substantially less in the recall challenge, whereas there was a great difference in the frequency of IL-10-producing cells in control animals during acute infection – again, this could be reflective of the kinetics of Tr1-like cell activation and expansion; third, the activation of Tr1-like cells may be context-specific *in vivo* and there could be a threshold of inflammation to trigger Tr1 cell activation, such as when excessive tissue damage is present; and fourth, the Tr1 response during primary infection could contribute to tolerogenic memory *via* limiting the formation of pathogenic Th2 resident memory cells, and depletion of Tr1-like cells immediately prior to re-challenge would not affect this phenomenon. Further efforts are required to determine whether Tr1 cells contribute to tolerogenic memory in allergy models. In the context of influenza vaccination, a population of lung-resident memory Tr1 cells are induced and further expand upon re-challenge, though a protective role for Tr1 memory cells was not assessed as their presence was an unexpected finding (43). In other instances, a role for memory Tr1 cells in promoting tolerance has been demonstrated. For instance, gliadin-specific Tr1 cells exist in the intestinal mucosa of celiac disease patients during disease as well as in the absence of cognate antigen (44),. These Tr1 cells responded to cognate antigen and robustly secreted IL-10. The longevity of Tr1 cells has also clearly been demonstrated in humans, both in the SCID patient where Tr1 cells were first discovered to be contributing to long-term tolerance (3) but also in patients treated with a Tr1-enriched cell therapy product for the prevention of graft-versus-host disease (GvHD) in conjunction with allogeneic stem cell transplantation to treat high-risk myeloid malignancies (14). In fact, TCR sequencing of a Tr1-enriched cell therapy product before infusion and of peripheral CD4^+^ T cells at various timepoints post-infusion revealed that Tr1 clones which were present at infusion could still be detected in circulation one year later (14). Current data suggest that Tr1 cells indeed contribute to tolerogenic memory, though more efforts should focus on mechanisms and kinetics of tolerogenic memory responses elicited by Tr1 cells.

The generation of Tr1 cells *in vivo* likely occurs through multiple mechanisms involving presentation of both autoantigens and persistent foreign antigens accompanied by signaling from APCs (10, 28, 29, 45–47). There is substantial evidence demonstrating that IL-10-producing APCs promote Tr1 development *in vivo*. The first indirect evidence came from the SCID patient in which Tr1 cells were discovered; monocytes from this patient highly expressed IL-10 mRNA (3). Indeed, IL-10^+^ monocytes have been demonstrated to induce a protective Tr1-like population *in vivo* during viral infection (48). Other IL-10-producing APCs such as IL-10-producing tolerogenic dendritic cells (DC-10), tolerogenic dendritic cells expressing the scavenger asialoglycoprotein receptor (ASGPR), and macrophages play important roles in tolerance *in vivo* by promoting Tr1 differentiation (46, 49, 50). Despite the prominent role of IL-10 which was clearly demonstrated in Tr1 differentiation, there were cases in which Tr1 cells were differentiated in the absence of exogenous IL-10 due to non-classical co-stimulation such as through CD2, CD55, ICOS, or B7H1 (51–54). However, the extent to which these pathways contribute to Tr1 development *in vivo* remains uncertain. Finally, co-stimulation through CD46 has also been described to induce Tr1-like cells (22, 55–57), but these are likely IL-10-producing Th1 cells instead of *bona fide* Tr1 cells [reviewed in (21)]. While distinct, it is important to mention that IL-10-producing Th1 cells can also contribute to peripheral tolerance. Irrespective of its role in inducing Tr1 differentiation, murine and human Tr1 cells require IL-10 signaling to function (54, 58). Beyond IL-10, other cytokines have been implicated in Tr1 differentiation as well. IFN-α complements IL-10 to promote Tr1 cells *in vitro* (59). Similarly, induction of functional Tr1 cells through co-stimulation with B7H1 required IFN-γ (54). This is in line with a recent report which suggested IFN-γ signaling was vital for Tr1 cell formation *in vivo* (60), though this remains to be seen in human. Extensive studies have been carried out on the role of IL-27 in regulating IL-10 and promoting Tr1 cell differentiation, but this has almost exclusively been performed in mouse models (61, 62). It was recently reported that IL-27 had only modest effects on IL-10 production in human CD4^+^ T cells (63). However, IL-27 promoted IL-10 production by Tr1-like cells overexpressing the transcription factor, EOMES (11). Accordingly, the effect of IL-27 on human Tr1 cell induction remains poorly characterized. Similar to IL-27, TGF-β contributes to murine Tr1 cell differentiation (61) but data from experiments with human cells do not support a role for TGF-β signaling in Tr1 differentiation (59, 64). Beyond these extensively studied cytokines, other soluble mediators such as melatonin can induce Tr1-like cells (37, 65). Taken together, the existing data strongly suggest that Tr1 cells differentiate following stimulation by tolerogenic APCs in the presence of IL-10 with possible roles for non-conventional co-stimulation and other soluble mediators. With recent advances in epigenetic tracking and gene editing of human CD4^+^ T cells (66–68), it is likely that the biology governing Tr1 cell differentiation will become more transparent.

**Figure 1:**
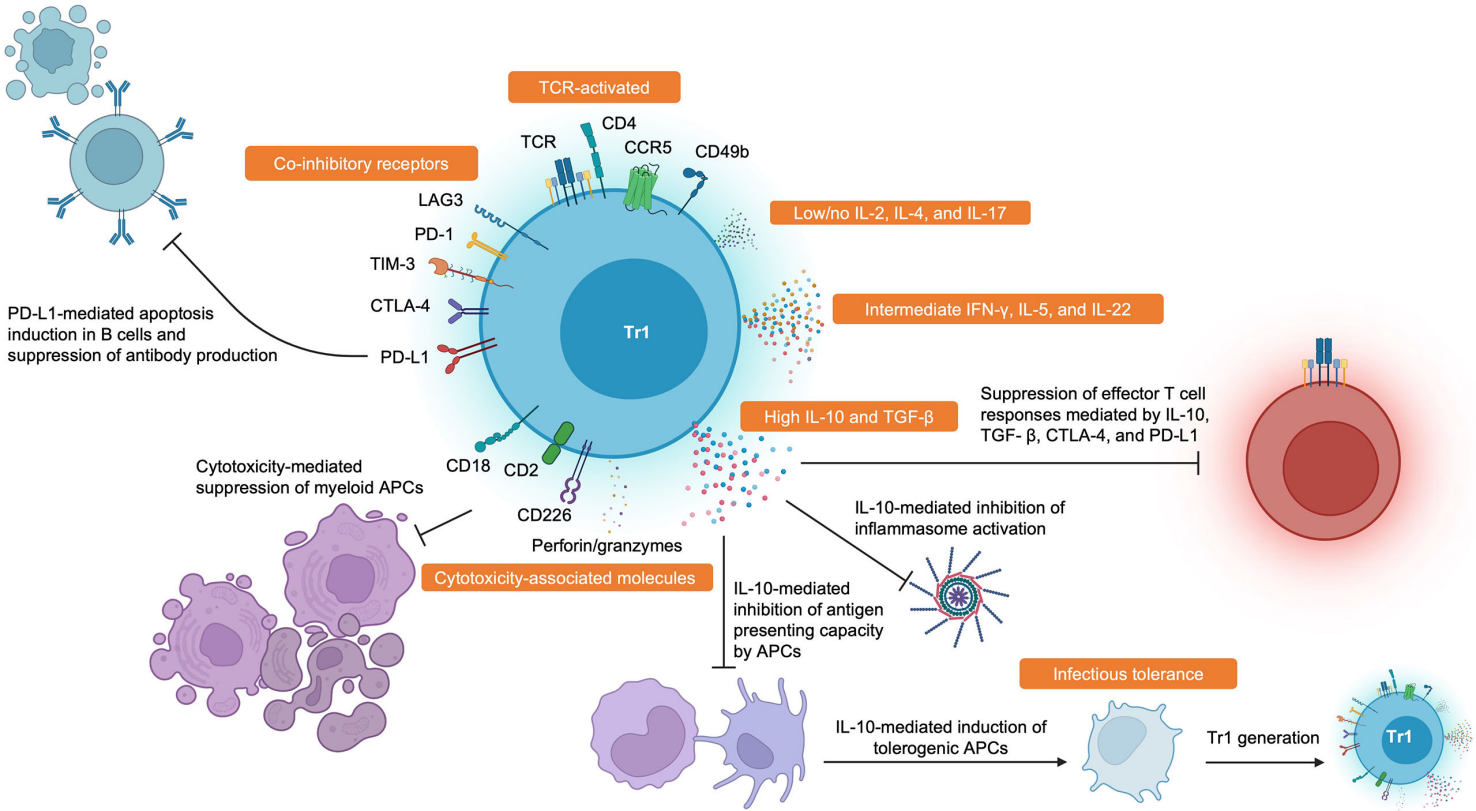
Hallmark Features and Functions of Tr1 Cells. Tr1 cells display high surface expression of CD49b, CCR5, CD2, CD18, CD226, and many co-inhibitory receptors such as LAG-3, PD-1, TIM-3, CTLA-4, and PD-L1. Tr1 cells characteristically secrete large amounts of IL-10 and TGF-β, intermediate amounts of IFN-γ and IL-5, and little to no IL-2 and IL-4. Tr1 cells have also been recently described to produce IL-22. Tr1 cells can suppress pathogenic immune responses through various mechanisms such as impairing antibody production by B cells, inducing PD-L1-mediated apoptosis in B cells, direct cytolysis of myeloid antigen presenting cells, reducing antigen presenting capacities by antigen presenting cells, induction of infectious tolerance, and direct inhibition of effector T cell responses through cytokine- and contact-dependent mechanisms. Created with BioRender.com.

Ultimately, the field suffers from the lack of a universal definition of Tr1 cells. Based on the wealth of data available, we consider Tr1 cells to be memory CD4^+^ T cells that suppress pathogenic immune responses in a TCR-dependent manner. They are FOXP3^-^ and have high expression of co-inhibitory receptors in addition to CD49b, CCR5, CD2, CD18, and CD226. They produce high amounts of IL-10 and TGF- β, intermediate amounts of IFN- γ and IL-5, and little to no IL-2, IL-4, and IL-17. Tr1 cells also secrete IL-22 which could be important in tolerance by mediating tissue repair (69, 70), and this could be another useful cytokine to identify Tr1 cells. Activated Tr1 cells are also cytotoxic and can eliminate myeloid cells through the secretion of perforins and granzymes. These criteria are summarized in **Figure 1**.

## Tr1-mediated tolerance in health

Tr1 cells are present in the circulation of healthy individuals (27) and also reside in tissues such as healthy colon (30, 31) and tonsils (15). Additionally, DC-10 cells were found in human decidual tissue where they are poised to give rise to Tr1 cells during pregnancy (71), and a population of Tr1-like cells was recently found in human decidual tissue, thereby suggesting Tr1 cells may contribute to fetal-maternal tolerance in an IL-10-dependent manner (72). Tr1 cells isolated from peripheral blood were activated in response to persistent antigens of self and foreign origin but were not activated in response to vaccine-associated antigens (28). In one such instance, islet-specific Tr1 cells were identified in non-diabetic individuals and were able to suppress antigen-specific effector T cell responses by eliminating APCs (10), thus representing a way Tr1 cells in health may prevent the onset of diabetes. Tr1 cells can be specific for many foreign antigens, but a substantial population is induced in the periphery following exposures to antigens associated with commensal microbes (29). Findings from IL-10-deficient mice revealed the importance of tolerance to commensal bacteria, as they only developed colitis when colonized with intestinal microbes (73). Much of what we know about Tr1 cells in health comes from studies correlating the frequency of Tr1 cells in healthy donors to those with inflammatory and autoimmune diseases. Intriguingly, the frequency of Tr1 cells amongst IL-10-producing cells is substantially reduced in ulcerative colitis (UC) and Crohn’s disease (CD) patients (30, 31). Desmoglein-3-specific Tr1 cells were found more frequently in healthy donors than in patients with pemphigus vulgaris, suggesting a protective role for Tr1 cells in prevention of the disease (45). Likewise, Tr1 cells were found more frequently in peripheral blood and synovial fluid of healthy donors and osteoarthritis patients than in rheumatoid arthritis (RA) patients, suggesting a protective role in the development of RA (74). Indeed, the frequency of Tr1 cells and Th1 cells at the site of inflammation was inversely correlated in RA patients. Taken together, these data indicate that Tr1 cells in healthy individuals protect against the development of autoimmune and inflammatory disease.

## Tr1 cells and tolerance in autoimmune disease settings

AIDs are a group of more than 80 disorders in which autoreactive immune cells are unable to distinguish self from non-self, resulting in highly inflammatory immune responses directed toward tissues containing the antigen. Globally, it is estimated that 3-5% of people have an AID and the incidence is increasing (75). In patients suffering from AIDs, regulatory T cells, including Tr1 cells, are unable to prevent this break of tolerance in affected tissues. For many of the AIDs, the antigens that are targeted by the host immune system are known and therefore antigen-specific Tr1 cell therapies can be designed. Such therapies could be a promising alternative to the current standard of care since general immunosuppressive drugs and biologicals may possibly result in increased susceptibility to infectious diseases. More recently, autoantigen-containing nanoparticles (NPs) have shown promising results to induce tolerance *in vivo* and the use of these biomaterials avoid high standards of manufacturing for cell therapies. In this section, we will focus on the role of Tr1 cells in T1D, MS, and other AIDs and how they can be applied to cure patients. The roles of Tr1 cells in various autoimmune and inflammatory diseases are summarized in **Table 1**.

**Table 1:** Overview of the role of Tr1 and IL-10-producing CD4^+^ T cells in AIDs and IBD.

Disease	Cell identity	Model	Major finding	Reference
***T1D***	FOXP3^-^ IL-10-producing CD4^+^ T cells	NOD BDC2.5 TCR-transgenic and NOD/SCID mice	Intestinal FOXP3^-^ IL-10-producing CD4^+^ T cells can protect from T1D	76
	IL-10-producing CD4^+^ T cells	NOD and NOD/SCID mice	Combination of rapamycin and IL-10 reduces incidence of diabetes	77
	GAD65-specific IL-10-producing CD4^+^ T cells	GAD65-immunized NOD and NOD/SCID mice	Protection from T1D in adoptive transfer experiments	78
	Antigen-specific IL-10-producing CD4^+^ T cells	Immunized NOD and NOD/SCID mice	Antigen-containing PLG nanoparticles protect from T1D	79
	CD49b^+^LAG-3^+^ Tr1 cells	Immunized NOD and NOD/SCID mice	IGRP- and 2.5mi/IA^g7^ nanoparticles expand pre-existing Tr1 cells and protect from T1D	80
	Islet-specific IL-10-producing CD4^+^ T cells	Patient PBMCs	Higher frequency in healthy donors	81
	Islet-specific IL-10-producing CD4^+^ T cells	Patient PBMCs	Higher frequency in first degree relatives	82
	Insulin-specific IL-10-producing CD4^+^ T cells	Patient PBMCs	Higher in patients that have better future glucose control	83
	IGFR-specific IL-10-producing CD4^+^ T cells	Patient PBMCs	Higher frequency in juvenile T1D compared to adult T1D	84
	IL-10-producing CD4^+^ T cells	Patient PBMCs	Increase in IL-10-producing CD4^+^ T cells upon anti-CD3 treatment	85
	Proinsulin-specific IL-10-producing CD4^+^ T cells	Proinsulin-immunized patient PBMCs	Increase in IL-10 production and decrease in insulin dependency	86
	IGRP-specific CD49b^+^LAG-3^+^ Tr1 cells	IGRP-immunized NSG mice reconstituted with DR4 patient PBMCs	Induction of IGRP-specific CD49b^+^LAG-3^+^ Tr1 cells	80
***MS***	OVA-specific IL-10-producing CD4^+^ T cells	OVA-immunized MSCH-induced EAE mice	OVA-specific protection from EAE development	87
	IL-10-producing CD4^+^ T cells	MOG-induced EAE mice	IL27-pulsed DCs upregulate IL-10 production by CD4^+^ T cells for protection from EAE development	88
	CD49b^+^LAG-3^+^ Tr1 cells	MOG-induced EAE mice	Protection from EAE development independently of IL-10	89
	IL-10-producing CD4^+^ T cells	MOG-induced EAE mice	Melatonin induces IL-10-producing CD4^+^ T cells to protect from EAE development	65
	CD49b^+^LAG-3^+^ Tr1 cells	PLP-induced EAE mice	PLP- and MOG-containing pMHCII nanoparticles protect from EAE development	80
	IL-10-producing CD4^+^ T cells	PLP- and MOG-induced EAE	MOG- and AhR-containing NLPs protects from EAE development	90
	CD46-induced IL-10-producing CD4^+^ T cells	Patient PBMCs	Lower frequency in MS patients	91
	CD46-induced IL-10-producing CD4^+^ T cells	Patient PBMCs	Altered glycosylation of CD46 in MS patients	57
***SLE***	CD46-induced IL-10-producing CD4^+^ T cells	Patient PBMCs	Lower frequency in SLE patients	24
	IL-10-producing IL7R^-^ CD4^+^ T cells	Patient PBMCs	Decreased ability in limiting autoantibody production by B cells	16
	IL-10-producing CXCR5^-^CXCR3^+^PD-1^high^ CD4^+^ T cells	Patient PBMCs	CXCR5^-^CXCR3^+^PD-1^high^ CD4^+^ T cell-derived IL-10 can stimulate autoantibody production	92
***Arthritis***	Collagen type II-specific IL-10-producing CD4^+^ T cells	Collagen type II-immunized DBA/1 mice	Protection from arthritis development	93
***Psoriasis***	CD49b^+^LAG-3^+^ Tr1 cells	Patient PBMCs and skin biopsies	Presence of Tr1 cells inversely correlated with disease severity	94
***Celiac disease***	Gliadin-specific IL-10-producing CD4^+^ T cells	Patient intestinal T cells	Gliadin-specific suppression of effector T cells	44
	Gliadin-specific IL-10-producing CD4^+^ T cells	Patient PBMCs and intestinal T cells	Multi-epitope gliadin extract induces tolerance in celiac disease patients	95
***IBD***	OVA-specific IL-10-producing CD4^+^ T cells	CD4^+^CD45RB^high^ adoptive transfer mice	Prevention of colitis in recipient mice	4
	IL10-deficient CD4^+^CD45RB^low^ T cells	CD4^+^CD45RB^high^ adoptive transfer mice	Protection from colitis is dependent on IL-10	96
	IL-10-producing CD4^+^ T cells	CD4^+^CD45RB^high^ adoptive transfer mice	Prevention of Th17-mediated colitis	97
	IL-10-producing CD4^+^ T cells	DSS-induced colitis mouse model	GSK-J4-mediated induction of tolerogenic DCs to promote IL-10 production by CD4^+^ T cells	98
	IL-10-producing CD44^+^CD4^+^ T cells	TNBS-induced colitis mouse model	MSCs induce expansion of IL-10-producing CD44^+^CD4^+^ T cells	99
	Intestinal IL7R^-^CCR5^+^PD-1^+^ Tr1 cells	Patient intestinal T cells	Decreased IL-10 production	30
	IL-10-producing OVA-Treg	Patient PBMCs	Amelioration of disease in treated patients	100
	CD49b^+^LAG-3^+^ Tr1 cells	Patient PBMCs and colon biopsies	Decrease of myeloid-derived inflammatory cytokines and production of IL-22 and mucin to promote barrier integrity	69

### Tr1 cells in T1D

T1D is characterized by a mainly Th1-mediated inflammatory reaction targeted towards insulin-producing β-cell islets in the pancreas, resulting in hyperglycemia and many co-morbidities. Interestingly, the HLA-DRB1*0401 haplotype is associated with development of multiple AIDs, including T1D and MS. T1D patients display a shift towards the presence of proinflammatory IFN-γ-producing Th1 cells while in healthy donors a strong bias towards protective IL-10 production can be observed upon islet peptide stimulation (81). Moreover, first degree relatives of T1D patients show a similar frequency of IFN-γ- and IL-10-producing T cells using ELISPOT assays although their T cells produce more IL-10 spot-forming units (SFUs). This indicates that, regardless of IFN-γ secretion, the ability of T cells to produce sufficient levels of IL-10 is an important factor for tolerance to pancreatic peptides (82). The idea that Tr1 cells, as most potent producers of IL-10, are important for T1D protection is further illustrated by the correlation of IL-10-producing CD4^+^ T cells at disease onset and future blood glucose levels (83). Interestingly, patients suffering from juvenile T1D have more protective antigen-specific Th2/Tr1 clones compared to inflammatory Th1 clones, which is in stark contrast to adult T1D patients (84). Early *in vivo* studies showed that anti-CD3 treatment induces tolerance in non-obese diabetic (NOD) mice, but this approach was largely ineffective in humans and was often accompanied by cytokine release syndrome caused by the crosslinking of the anti-CD3 FcR with autoreactive T cells (101). Therefore, Herold et al. designed an anti-CD3 antibody having an FcR with reduced binding capacity which resulted in an IL-10-mediated induction of tolerance in T1D patients (85). Interestingly, intestinal Tr1 cells may be an important source of these *in vivo* induced protective IL-10-producing CD4^+^ T cells as Tr1 cells that arise in the small intestine protected NOD/SCID mice from T1D development upon co-transfer with diabetogenic BDC2.5 T cells (76). To further support this finding, rectally injected Tr1 cells were able to migrate to the pancreas to prevent T1D onset. Tr1 cells isolated from the gut mucosa, the main site of Tr1 induction, are thus able to confer protection to autoreactive T cells in a T1D context and possibly other AIDs (76). Since the use of anti-CD3 antibodies resulted in undesired side effects, other approaches for inducing tolerance in T1D were investigated. Rapamycin, known to induce and maintain FOXP3^+^ Tregs, was used in combination with IL-10 to induce tolerance to insulin-producing pancreatic islets in the NOD mouse model (77). In these mice, splenic CD4^+^ T cells produced larger amounts of IL-10 which thus could be the cause of a significant delay in diabetes onset. Furthermore, Tr1 cells isolated from the spleens of these mice suppressed the proliferation of diabetic splenocytes and transfer of pancreatic islets from treated mice to NOD/SCID mice showed that these T cells were indeed protective. Injection of IL-10 alone did not protect NOD mice from diabetes which might indicate that rapamycin first needs to reduce effector T cell proliferation before IL-10 is able to maintain a stable Tr1 cell population, as illustrated by a synergistic effect of rapamycin and IL-10 treatment. These studies show that both anti-CD3 treatments and the use of rapamycin and IL-10 might induce tolerance mediated by polyclonal Tr1 cells. Importantly, however, these *in vivo* induced polyclonal Tr1 cells might result in infectious tolerance and therefore more recent *in vivo* studies investigated the potential of autoantigens to generate antigen-specific Tr1 cells for peptide immunotherapy. Chen et al. utilized the 65 kDa glutamic acid decarboxylase (GAD65) to immunize NOD mice to establish antigen-specific Tr1-mediated tolerance (78). Co-transfer of diabetogenic T cells and Tr1 clones into lymphopenic NOD/SCID hosts resulted in protection from T1D in an IL-10-dependent manner. A study in T1D patients illustrated that immunotherapy using the immunodominant proinsulin peptide resulted in a decrease in insulin dependency which thus could be attributed to tolerance induction although the precise mechanism was not elucidated (86). Rather than injecting soluble peptides, NPs capable of encapsulating disease-causing peptides are a valuable alternative for treating AID patients. Using NPs harboring multiple cognate diabetogenic peptides, Prasad et al. demonstrated that this approach induced tolerance upon adoptive transfer of T1D-specific proinflammatory T cells (79). Although Foxp3^+^ Tregs were increased in both the spleen and the pancreas of host mice, splenocytes produced increased levels of IL-10 which might implicate a possible role for CD49b^+^LAG-3^+^ Tr1 cells. Another study exploiting the same approach found similar results using NPs containing insulin peptides, which is the cognate antigen of the BDC2.5 TCR-transgenic mouse model. Adoptive transfer experiments again resulted in the protection from T1D in an antigen-specific manner as illustrated by tetramer staining on antigen-specific regulatory T cells (102). The formal proof that tolerogenic Tr1 cells are induced *in vivo* upon administration of antigen-containing NPs came from a study in which multiple diabetogenic peptides were loaded onto recombinant MHC-II (pMHC-II) (80). *G6pc2*^-/-^ NOD mice treated with these pMHC-II NPs showed an expansion of pre-existing Tr1 cells since only BDC2.5 memory T cells were protective upon adoptive transfer of NOD/SCID mice whereas their naïve counterparts were not. Interestingly, from a translational approach, viral challenge of pMHC-II NP-treated mice resulted in protection from both T1D and viral infections. Adoptive transfer experiments using DR4-specific peripheral blood mononuclear cells (PBMCs) isolated from T1D patients also resulted in an increase in diabetes-free survival upon co-injection of pMHC-II NPs. These results highlight that antigen-specific Tr1 cells induced by pMHC-II NPs can mediate tolerance while still allowing a potent immune response towards pathogens.

### Tr1 cells in MS

Tr1 cells also play a key role in MS. MS is an AID characterized by an excessive inflammatory immune response in which autoreactive T cells target the central nervous system (CNS) white matter. Early reports investigating the role of Tr1 cells in MS pathogenesis showed that an alteration in the expression of the isoforms of the co-stimulatory molecule CD46, important for a shift from a proinflammatory to an anti-inflammatory immune response, resulted in a reduced ability to induce IL10-producing CD4^+^ T cells in MS patients (91). Moreover, a follow-up study showed that impaired glycosylation of CD46 upon TCR stimulation in MS patients prevented the shift from IFN-γ-producing T cells toward IL-10-secreting T cells (57). Barrat et al. were among the first to show that IL-10-producing CD4^+^ T cells were able to protect from experimental autoimmune encephalitis (EAE), a mouse model of MS (87). Here, the combination of vitamin D3 (VitD3) and dexamethasone (Dex), both known to be immunosuppressive, enriched for IL-10-producing CD4^+^ T cells *in vitro*. Of note, these cells were presumably not a homogenous population of Tr1 cells as they did not produce IFN-γ which is a hallmark cytokine of *bona fide* Tr1 cells. To demonstrate the antigen-specificity of these IL-10-producing CD4^+^ T cells, EAE mice were first intracranially injected using OVA and subsequently injected with OVA-specific VitD3/Dex-induced Tr1-like cells. These cells were able to significantly reduce both the EAE clinical score and T cell infiltration in the CNS in an antigen-specific manner. Another study investigated the potential of IL27-pulsed dendritic cells (DCs) to induce tolerance in an EAE mouse model (88). These IL27-primed tolerogenic DCs were able to upregulate IL-10 secretion in Tr1-polarizing cultures using naïve 2D2 CD4^+^ T cells, a population isolated from TCR-transgenic mice specific for myelin oligodendrocyte glycoprotein (MOG). Vaccination of EAE mice with IL27-conditioned DCs showed to improve EAE although the specific role of Tr1 cells was not addressed in these experiments (88). Interestingly, however, *Il27ra* knockout mice showed an increase in EAE clinical score and a decreased production of IL10-producing splenic CD4^+^ T cells which might indicate a loss of Tr1 cells due to the absence of tolerogenic DCs. Of note, although the impact of IL-27 signaling on the induction of *IL10* expression through Blimp-1 and c-Maf during Tr1 differentiation has been demonstrated in mice, similar results have not been observed in human (35). Evidence that Tr1 cells are instrumental for tolerance in EAE came from a study that immunized mice using the autoantigen MOG in the presence of retinoic acid and IL-2 as adjuvants (89). While these mice had an increase in IL-10-producing CD49b^+^LAG-3^+^ Tr1 cells that protected from EAE progression due to a decrease in Th17 and Th1 cells, FOXP3^+^ Treg abundance was not altered. Of note, the same experiments were also performed in *Il10*^-/-^ mice which showed that although Tr1 cells mainly inhibit immune responses *via* IL-10 secretion, these mice were still protected from EAE. This finding might indicate that, in MS, Tr1 cells may contribute to immunomodulation *via* alternative mechanisms or they might instruct other tolerogenic cells to mediate tolerance but studies in human should be performed. Another interesting study showed that melatonin has the potential to reduce disease severity in MS patients as melatonin is an important mediator of disease activity resulting from seasonal changes. Indeed, administration of melatonin in multiple EAE models increased IL-10 production by Tr1 cells while Th17 differentiation was inhibited and thus the use of melatonin could be an interesting avenue for tolerance induction (65). From a translational perspective, the use of NPs in EAE also proved to protect mice from disease in an antigen-specific manner. In an early report, myelin epitope-containing microparticles prevented EAE development *via* uptake through the macrophage receptor with collagenous structure (MARCO) which eventually resulted in a reduction of pathogenic IL-17^+^ and IFN-γ^+^ CD4^+^ T cells. However, the possible impact of Tr1 cells to restore balance in these mice was not investigated (103). Next to the use of pMHC-II NPs to treat T1D in mice, Clemente-Casares et al. also investigated the use of pMHC-II NPs containing MOG to prevent EAE progression (80). Here, the authors found that antigen-specific CD49b^+^LAG-3^+^ Tr1 cells were increased in treated mice with concomitant absence of demyelination of white matter in the cerebellum compared to untreated controls. Finally, Kenison et al. used nanoliposomes (NLPs) that contain both the MOG peptide and an aryl hydrocarbon receptor (AhR) agonist to establish tolerance in EAE mice *via* induction of tolerogenic DCs (90). Indeed, these NLPs were able to generate antigen-specific IL-10-producing CD4^+^ T cells which mediated the reduction of IL-17^+^ and IFN-γ^+^ pathogenic T cells. However, the presence of Tr1 cells by validation of CD49b and LAG-3 expression was not investigated.

### Tr1 cells in other AIDs

In addition to T1D and MS, Tr1 cells also promote tolerance in other AIDs such as systemic lupus erythematosus (SLE), RA, psoriasis, and celiac disease. Similar to what has been observed in MS patients, patients with lupus nephritis, which is a major cause of SLE, the CD46 co-stimulatory pathway was affected and prevented the conversion of Th1 to IL-10-producing Th1 cells (24). Interestingly, it has also been shown that although Tr1 cells are more abundant in the peripheral blood compared to healthy donors, Tr1 cells isolated from SLE patients are unable to restrict IgG production by B cells which indicates that Tr1 cells play a crucial role in limiting autoantibody production by autoreactive B cells (16). This finding was further supported by experiments in which co-transfer of B cells and Tr1 cells in OVA-immunized *Rag1*^-/-^ mice resulted in a decrease of OVA-specific IgG production of the transferred B cells. However, their protective role in MS might not be strictly attributable to IL-10-mediated tolerance induction since a recent publication demonstrated that IL-10 produced by CXCR5^-^CXCR3^+^PD-1^high^ CD4^+^ T cells stimulates antibody production by autoreactive B cells (92). Also, in a mouse model of RA, there was evidence that Tr1 cells are key to instruct tolerance towards RA autoantigens (93). This work demonstrated that Col-Tregs (collagen type II-specific Tr1 cells), induced *ex vivo* by the incubation of collagen type II TCR-specific splenocytes with IL-10 and collagen type II antigen, resulted in the generation of RA-specific Tr1 cells capable of reducing erosion, cell infiltration, B cell response and hyperplasia in the paws from arthritic mice. These Col-Tregs also produced IFN-γ which might indeed indicate that these cells are enriched for *bona fide* Tr1 cells. In psoriasis, Tr1 cells might also play an important role in preserving tolerance. Kim et al. found that psoriasis patients, in contrast to SLE patients, had less CD49b^+^LAG-3^+^ Tr1 cells in the peripheral blood and this observation correlated to disease severity (94). Furthermore, the authors illustrated that Tr1 cells were present in non-lesional skin while in psoriatic lesions there was an increase in infiltrating T cells and here Tr1 cells could not be detected, leading to the idea that the absence of Tr1 cells allows Th1-mediated inflammation of the skin. Finally, Tr1 cells are also implicated in celiac disease, a condition characterized by the intolerance to gluten in the gut of affected individuals. An important study by Gianfrani et al. generated gliadin-specific IL-10-producing cells by culture of cells from patient-derived mucosal explants on a PBMC feeder in the presence of the gliadin antigen and IL-10 (44). These clones produced large amounts of IL-10 and IFN-γ and, importantly, were anergic to gliadin-specific stimulation which is an important feature of Tr1 cells. Moreover, these gliadin-specific IL-10-producing clones inhibited the proliferation of responder T cells in an antigen-specific fashion. Recently, a double-blinded placebo-controlled clinical trial using TAK101, a NP containing a multi-epitope gliadin extract, showed to induce tolerance with a reduction in pathogenic effector T cells in patients which paves the way for novel and less toxic therapies for celiac disease (101).

In summary, it has been shown using *in vivo* models that Tr1 cells are critical to protect from T1D and MS development while their role in other AIDs has not been fully investigated. IL-10 plays a prominent role in establishing tolerance in SLE, RA, psoriasis, and celiac disease which might indicate that *bona fide* CD49b^+^LAG-3^+^ Tr1 cells are involved but this requires more investigation. Induction of antigen-specific tolerance in AIDs, either *via* cell therapy or other modalities, would transform the therapeutic management of these diseases.

### The role of Tr1 cells in IBD

IBD comprises a group of severe diseases of the gastrointestinal (GI) tract in which an autoinflammatory immune response is mounted against enteric antigens in genetically susceptible hosts. Over the past few decades, the prevalence of IBD, including very early inflammatory bowel disease (VEO-IBD), CD, and UC, has substantially increased mainly in western and industrialized countries (104). IBD is a complex and multifactorial disease mainly characterized by severe colitis and weight loss with important co-morbidities. In the intestinal microenvironment of IBD patients, many key players of the immune system target otherwise harmless enteric and environmental antigens in which both FOXP3^+^ Tregs and Tr1 cells have lost their ability to suppress these undesired immune responses. Here, we focus on the role of Tr1 cells in IBD and their immunosuppressive abilities *via* the production of IL-10.

IL-10-producing cells are mainly found in the in the lamina propria of the GI tract (105–107). Although Tr1 cells produce high amounts of IL-10 and have broad immunosuppressive properties, it recently has been demonstrated using adoptive transfer of IL-10^+^FOXP3^-^ Tr1 cells in lymphopenic hosts that there is substantial spatial heterogeneity among Tr1 cells. For instance, IL-10^+^FOXP3^-^ splenic Tr1 cells are less protective against colitis development in contrast to their intestinal counterparts (31). The heterogeneity in IL-10-producing cells is further reflected in single cell studies using gut biopsies from IBD patients in which only a small cluster of IL-10-producing lymphocytes represents *bona fide* Tr1 cells, characterized by high expression of multiple co-inhibitory receptors (31). In general, Tr1 cells isolated from IBD patients produce less IL-10 than their FOXP3^+^ Treg counterparts upon *ex vivo* stimulation, which highlights that loss of Tr1-specific IL-10 is an important mediator of IBD (23, 30). Importantly, it has also been shown that proinflammatory cytokines implicated in IBD, such as IL-23 and IL-1β, cause this loss of IL-10 secretion by Tr1 cells which illustrates that the proinflammatory microenvironment present in IBD patients prevents Tr1-mediated tolerance (30).

The first formal evidence of the protective role of IL-10-producing Tr1 cells was provided by Groux et al. Here, it was demonstrated that clonally expanded OVA-specific Tr1 cells were able to prevent colitis in the CD4^+^CD45RB^high^ adoptive transfer mouse model in an antigen-specific manner (4). Of note, these findings might also indicate that Tr1 cells could potentially provide bystander suppression to enteric antigens (108). The crucial role of IL-10 in gut tolerance was further shown by the inability of *Il10*-deficient CD4^+^CD45RB^low^ T lymphocytes, containing both Tr1 and FOXP3^+^ Treg cells, to restore homeostasis in the bowels of mice that were injected using inflammatory CD4^+^CD45RB^high^ T cells (96). Importantly, in the same study, injections of IL-10 did not ameliorate IBD symptoms which highlights the need for T cell-derived IL-10 to induce long-term tolerance in IBD patients (96). Studies using this CD4^+^CD45RB^high^ adoptive transfer model also demonstrated the role of gut microbiota in shaping intestinal tolerance since CD4^+^CD45RB^high^ T cells isolated from germ-free mice were unable to induce colitis in hosts (109). Interestingly, these adoptive transfer studies in mice are performed using polyclonal T cells which indicates that these cells might induce *de novo* antigen-specific Tr1 and Treg cells for long-term tolerance in IBD. Since the CD4^+^CD45RB^low^ subset contains both Tr1 cells and FOXP3^+^ Treg cells, FOXP3^+^ Treg cells may also mediate tolerance *via* IL-10 and this has been elegantly shown by a *Foxp3*^Cre^*Il10*^LoxP^ mouse model that indeed showed intestinal inflammation (110). Interestingly, the intestinal inflammation observed in these *Foxp3*^Cre^*Il10*^LoxP^ mice is very similar to the gut pathology of *Il10*^-/-^ mice while inflammation of the skin is only seen in the latter which might indicate that homeostasis in the skin is mainly mediated by Tr1 cells and myeloid cells, such as macrophages and DCs (111). Combined, these studies thus show that although Tr1 cells produce large amounts of IL-10, the total level of IL-10 produced by intestinal T cells needs to reach a certain threshold to maintain tolerance. This has been further demonstrated by a study in which both FOXP3^+^ Tregs and Tr1 cells control Th17-mediated colitis in an IL-10-dependent manner as pathogenic Th17 cells expressing a dominant negative form of the IL-10 receptor were unresponsive to IL-10-mediated suppression (97). In addition, IL-10 also plays a critical role in amplifying the immunosuppressive role of tolerogenic T cells as loss of the IL-10 receptor on both FOXP3^+^ Tregs and Tr1 cells results in the loss of suppression of pathogenic Th17 responses in the gut (58, 112). Here, the loss of IL-10 signaling in Tr1 cells only resulted in a decrease of IL-10 secretion while other immunosuppressive mechanisms such as CTLA-4 and Granzyme B function were unaffected. This finding thus indicates that IL-10 is crucial for both dampening the production of inflammatory cytokines by pathogenic T cells and for amplifying the immunosuppressive ability of Tr1 cells and FOXP3^+^ Tregs (58). However, one study showed that only injection of OVA-specific Tr1 cells, and not FOXP3^+^ Tregs, multiple weeks after colitis onset resolved intestinal inflammation which indicates that mainly Tr1 cells are able to expand *in vivo* to control inflammation and ongoing disease and ongoing disease (90).

Studies in human and mice have clearly shown that Tr1 cell therapy holds great promise as an alternative treatment capable of inducing long-term tolerance in the bowels of IBD patients. For instance, the use of autologous OVA-Tr1 cells has been tested in a phase I/IIa clinical trial with a maximum effect 5 weeks after administration but follow up studies were never performed (NCT02327221) (100). A more recent study by Cook et al. showed that polyclonal activation of CD4^+^ T cells isolated from the peripheral blood of IBD patients enabled the *ex vivo* expansion of IL-10-producing CD49b^+^LAG-3^+^ Tr1 cells (69). These clonally expanded Tr1 cells were able to reduce the secretion of the proinflammatory cytokines TNF-α and IL-1β by myeloid cells. In addition, these Tr1 cells not only secreted IL-22 which promoted barrier integrity of epithelial cell lines, but they were also able to induce mucin production by goblet cells in patient-derived gut organoids, both features that were not seen with expanded FOXP3^+^ Tregs (69). These preliminary results demonstrate that Tr1 cells, rather than FOXP3^+^ Tregs, might be promising as a novel cell therapy for IBD patients. Next to these *ex vivo* expanded Tr1 cells, tolerogenic DCs might also represent an interesting approach for resolving IBD. Administration of GSK-J4, a histone demethylase inhibitor, resulted in the retinoic acid-mediated induction of tolerogenic DCs in the dextran sulfate sodium (DSS) colitis mouse model with concomitant production of IL-10 by CD4^+^ T cells (98). However, the induction of CD49b^+^LAG-3^+^ Tr1 cells was not shown in this model and since GSK-J4 might affect other important pathways as a broad epigenetic modifier, the use of this compound might result in multiple undesired off-target effects (98). Finally, many clinical trials are now investigating the potential use of mesenchymal stem cells (MSCs) in reducing IBD severity. Using the 2,4,6-trinitrobenzenesulfonic acid (TNBS) colitis mouse model, it has been shown that MSCs are capable of expanding the existing Tr1 pool. However, co-expression of CD44 and IL-10 was used to define these putative Tr1 cells while the presence of both CD49b and LAG-3 was not shown (99).

As the most important source of IL-10 in the gut, Tr1 cells play a non-redundant role in the protection from IBD in human. Together with other genetic risk, the composition of the gut microbiome is an important factor in the onset and progression of IBD. IBD patients that have mutations in the *IL10* or *IL10R* genes suffer from very severe intestinal disease which indeed highlights that Tr1-mediated IL-10 signaling maintains tolerance to commensal microbiota in the gut. The enteric antigens that induce IBD have been extensively studied but the bacteria that are implicated might differ substantially between individuals. Nevertheless, future clinical trials investigating the use of polyclonal Tr1 alone or together with anti-inflammatory drugs might prove promising to induce long-term tolerance in the bowels of affected patients.

## The role of Tr1-induced tolerance in infectious disease settings

While it is very well established that Tr1-mediated tolerance confers protection in autoimmune contexts, the role for Tr1 cells in infectious disease is ill-defined. Shortly after their discovery, research began to address whether Tr1 cells are friend or foe in infectious disease, a context where the balance between inflammatory and anti-inflammatory responses is critical to permit pathogen clearance without excessive pathology. In a murine model of *Bordetella pertussis* infection, antigen-specific Tr1 cell clones were produced from lung-infiltrating T cells of infected mice (113). The resulting clones were suppressive *in vitro* and caused a small delay in viral clearance upon adoptive transfer in combination with Th1 cells. However, it was not demonstrated whether immunopathology was affected in this model. In contrast, a more recent paper demonstrated that Tr1 cells did not impair the immune response to acute viral infection (114). In this case, mice which were treated with Tr1 cells (LAG-3^+^ CD4^+^ T cells) to prevent allograft rejection were infected with lymphocytic choriomeningitis virus (LCMV), but viral clearance and generation of antigen-specific effector T cells was comparable to what was observed in non-transplanted/non-treated C57BL/6 mice. Consistent with this finding, human T cell populations containing allo-antigen-specific Tr1 cells proliferated effectively when challenged with foreign antigens and 3^rd^ party APCs (14, 64). We previously demonstrated that CD49b^+^LAG-3^+^ Tr1 cells were induced *in vivo* during *Nippostrongylus brasiliensis* infection, though their emergence in the lungs and mediastinal lymph nodes corresponded with the resolution of inflammation after pathogen clearance (27). Similarly, Tr1 cells were recently shown to be present within the lung during influenza infection in mice (43). Remarkably, Omokanye et al. demonstrated that Tr1 cells exist within the influenza-specific lung-resident memory T cell population 6 months post-vaccination, and the Tr1 cells clonally expanded following re-challenge. Despite the induction of influenza-specific Tr1 cells in this model, mice were able to survive lethal infection with low morbidity (115). While no functional experiments were performed to establish a role for the Tr1 cells in this model, the authors speculated that the Tr1 cells likely serve as a mechanism to limit pathology within the lung during acute infection, similar to what has been observed for FOXP3^+^ Tregs during influenza infection (116–120). In support of this hypothesis, recent evidence suggests a protective role of Tr1-like cells in murine gamma herpesvirus infection in which infection induced IL-10-producing monocytes within the lung that led to generation of Tr1-like cells (48). Though the cells were not referred to as Tr1 cells, their single-cell RNA sequencing revealed these cells had a Tr1-like transcriptional profile exemplified by high expression of *Gzmb, Gzma, Il10, Lag3, Maf, Ctla4, Id2, Nkg7*, and *Ccr5*. Upon monocyte depletion, these cells were absent and instead a population of cytotoxic CD4^+^ T cells persisted and elicited severe tissue damage within the lungs, suggesting a protective role of Tr1-like cells generated by IL-10-producing monocytes during acute infection. Overall, Tr1 cells do not seem to hinder effective immune responses needed for pathogen clearance during acute infections but on the other hand exert a protective role against excessive inflammation and immune activation induced by the pathogen.

While the study of Tr1 cells in acute infection is rather limited, significant efforts have aimed at determining whether Tr1-mediated tolerance plays a part in recurrent or chronic infections. One of the first reports on the topic examined PBMCs from patients infected with *Onchocerca volvulus* who developed patent infection instead of sterilizing immunity (121). Hyporesponsiveness to antigen was driven by IL-10 and TGF-β, and T cell clones from one patient revealed a Tr1-like cytokine profile. Surprisingly, antigen-specific Tr1-like cell clones could not be generated from previously infected patients who developed immunity. In another setting, the frequency of Tr1 cells correlated with disease progression in HIV-infected individuals, but a causative link was not established, and more data are needed to determine whether Tr1 cells contribute to HIV pathogenesis (122). The frequency of Tr1 cells was also associated with a severe form of paracoccidioidomycosis, but the frequency of Tr1 cells in these patients was only slightly elevated compared to healthy controls and patients with a mild form of chronic infection (123). Conversely, patients with an acute form of paracoccidiomycosis had a substantial increase in circulating Tr1 cells. In the context of chronic viral infection, patients with chronic hepatitis C virus (HCV) infection presented with elevated levels of serum IL-10 (124). Using T cells from these patients, HCV core-specific cell lines and clones were generated which adopted either Th1 or Tr1 cytokine profiles. While no functional studies were performed to demonstrate the ability of the Tr1 cell lines and clones to suppress HCV-specific immune responses, similar work provided more insight. PBMCs from HCV-infected patients produced substantial amounts of IL-10 upon stimulation with another HCV protein, nonstructural protein 4, and IFN-γ production was minimal, but IFN-γ production was rescued in the presence of an IL-10-neutralizing antibody (125). However, it was then demonstrated that IL-10 production largely came from monocytes in this model and the monocyte-derived IL-10 was sufficient to suppress DC maturation and T cell responses to polyclonal and antigen-specific stimulations. Nevertheless, these data suggest that HCV-derived proteins could elicit antigen-specific Tr1 differentiation *in vivo* which could potentially impair effector responses against HCV, though this remains to be demonstrated. Several reports have explored the hypothesis that Tr1 cells contribute to HCV recurrence following liver transplantation but a direct causative role for Tr1 cells has not been demonstrated (126–129).

The most cohesive body of literature surrounding the role of Tr1-mediated tolerance during infectious disease arose in the context of malaria infection. Protective immunity to malaria develops slowly and follows repeated infection [reviewed in (130)]. This form of immunity is often referred to as “clinical immunity” and it does not constitute a sterilizing immune response (131). Children living in malaria-endemic settings have a much higher probability of asymptomatic infection following repeated exposure. Thus, clinical immunity is defined by health despite parasitemia. Children repeatedly exposed to malaria in endemic settings develop a substantial proportion of antigen-specific IL-10^+^IFN-γ^+^ CD4^+^ T cells (132), prompting the question whether Tr1-mediated tolerance may contribute to the development of clinical immunity to *Plasmodium* infections. Multiple studies utilizing mouse models of malaria infection demonstrated a protective role of Tr1-like cells. One study utilizing *Plasmodium chabaudi chabaudi* demonstrated that engraftment of wildtype but not IL-10-deficient CD4^+^ T cells protected *Rag*^-/-^ mice from serious weight loss, hypothermia, and mortality following infection (133). A separate study using *Plasmodium yoelii* demonstrated that although *Rag*^-/-^ mice engrafted with WT as opposed to IL-10-deficient CD4^+^ T cells had increased parasitemia following infection, these mice experienced less severe weight loss and a modest improvement in survival duration (134). These two studies demonstrate, using separate mouse models, that CD4^+^ T cell-derived IL-10 promotes health while potentially inhibiting parasite clearance. However, Tr1-like cells may not influence parasitemia to the same degree as FOXP3^+^ Tregs, which significantly increased parasite levels in the blood of *Plasmodium yoelii*-infected mice (134, 135). Additionally, FOXP3^+^ Tregs provided insufficient protection against pathology during malaria infection, and IL-10 from effector CD4^+^ T cells sufficiently limited pathology even in the absence of FOXP3^+^ Tregs (133). Thus, compared to Tregs, Tr1-like cells provide superior protection against immune-mediated pathologies while seemingly inhibiting anti-parasite immune responses to a lesser degree. As the latter report (133) claimed that the major source of protective IL-10 came from effector CD4^+^ T cells, it should be carefully considered whether these studies were looking at true Tr1 cells or IL-10-producing Th1 cells, which are distinct [reviewed in (21)]. While one group suggested the cells in their model were Tr1 cells and not IL-10-producing effectors based on the absence of IFN-γ, IL-4, IL-13, and IL-17a mRNA expression (136), recent single-cell RNA sequencing experiments in a mouse model of malaria revealed an emergence of Tr1-like cells from the Th1 lineage, but the transcriptomic profile between Th1 cells and Tr1-like cells were nearly identical, with only two genes besides *Il10* being differentially expressed between the two cell subsets (137, 138). Indeed, it is well documented that effector T cells may upregulate IL-10 in inflammatory settings to self-limit inflammation [reviewed in (139)].

Beyond findings in the mouse, recent findings have shed light on the role of Tr1-like cells in human malaria. In endemic settings, the frequency of IL-10-producing CD4^+^ T cells correlated with the number of malaria exposures (132). In line with this finding, T cells were less proliferative when isolated from children with a higher incidence of infection compared to T cells isolated from children with less frequent infections. This proliferative defect was at least partially IL-10-dependent (132). Surprisingly, the frequency of Tr1-like cells in malaria-exposed children did not increase with age, while the frequency of malaria-specific Th1 cells did (140). Additionally, the frequency of IL-10^+^ CD4^+^ T cells correlated with protection against symptomatic infection and induction of asymptomatic infection (140), consistent with the hypothesis that Tr1 cells can protect against excessive pathology during infection and may contribute to clinical immunity. It was previously reported that the most suppressive population of IL-10-producing CD4^+^ T cells in mice are CD49b^+^, LAG-3^+^, and highly express co-inhibitory receptors such as TIM-3, TIGIT, CTLA-4, and PD-1 (31). A CTLA-4^+^PD-1^+^ population of CD4^+^ T cells was identified in the blood of patients who had recently travelled to malaria-endemic regions and became infected (141). These cells co-produced IFN-γ and IL-10 in an antigen-specific manner, but IL-10 production was not detected upon polyclonal stimulation. Conversely, the CTLA-4^+^PD-1^+^ cells suppressed effector T cell responses *in vitro* when stimulated with infected red blood cells or with anti-CD3/CD28, suggesting suppression elicited by these cells was not dependent on IL-10. Indeed, cell-cell contact was necessary for suppression. Notably, these cells proliferated in response to antigen, and they displayed rapid contraction when patients were treated with antimalarials, which is consistent with what was observed for IL-10-producing Th1 cells in a mouse model of *Plasmodium* infection (138). While the frequency of CTLA-4^+^ CD4^+^ T cells was elevated in patients with severe cerebral malaria, there was no correlation between the frequency of CTLA-4 or PD-1 expression and parasitemia (141). Whether these were Tr1 cells or a subset of effectors induced specifically in the context of malaria should be explored. Follow-up studies from this group demonstrated that both CD4^+^ T cells and CD8^+^ T cells from malaria-infected individuals adopted a Tr1-like phenotype and cytokine profile, illustrated by high expression of LAG-3 and co-inhibitory receptors accompanied by production of IL-10, IFN-γ, and granzyme B (142). While functional studies were not performed with the human cells, analogous cells were induced in a murine model of malaria infection (142). The murine LAG-3^+^ cells were suppressive irrespective of CD49b expression, and the frequency of LAG-3^+^ cells in various tissues dwindled to levels seen in naïve mice within days of anti-malarial administration, suggesting these were not long-lived memory cells. Unexpectedly, the co-inhibitory receptor-rich CD8^+^ T cells had enhanced antigen-specific cytotoxic function (142, 143). Based on the presently available evidence, it can be concluded that Tr1-like cells prevent symptomatic malaria infection by protecting against immune-mediated pathology, as demonstrated in mice and humans (133–135, 140). Whether the Tr1-like cells are *bona fide* Tr1 cells instead of Th1 cells expressing IL-10 remains to be seen, but regardless, the IL-10-producing CD4^+^ T cells in malaria limit disease-associated pathology and may contribute to clinical immunity.

Collectively, these data suggest that across different infection models and in both acute and chronic settings, Tr1 cells and Tr1-like cells limit immune-mediated pathology during infection. In some contexts, it is possible that Tr1-mediated tolerance could contribute to delayed pathogen clearance or favor recurrent infection, though more data are warranted to draw strong conclusions. Whether Tr1 cells inhibit pathogen clearance could also depend on the microenvironment governing their function during infection. Indeed, FoxP3^+^ Tregs exert different immunoregulatory functions at various stages of infection (119). Further studies should rigorously explore the role of Tr1 cells across the full duration of infection and in re-infection models to distinguish context-dependent roles of Tr1 cells during infection. This information will be vital as Tr1 cells receive more attention in clinical settings.

## Importance of Tr1 in the face of other regulatory T cells

Tr1 cells have a vital role in promoting peripheral tolerance. However, they are not the only regulatory cell involved in tolerance, as natural and inducible FOXP3^+^ Tregs and other emerging regulatory cell types undoubtedly contribute [reviewed in (144)]. This begs the question: why do we have Tr1 cells? They could exist as a fail-safe for when other regulatory cells are insufficient to limit an immune response. For instance, in immunodysregulation polyendocrinopathy enteropathy X-linked syndrome (IPEX) patients, deleterious mutations in *FOXP3* result in a failure of Tregs to suppress autoimmunity. Intriguingly, T cells from IPEX patients can differentiate into Tr1 cells (145), and increased frequencies of Tr1 cells correlate with diminished disease severity (146). Another reason Tr1 cells may exist is the broad antigen specificity Tr1 cells can acquire. FOXP3^+^ Tregs are generated in the thymus and recognize self-antigens, though the antigenic repertoire does not cover the entirety of self-antigens [reviewed in (147)]. Accordingly, Tr1 cells may fill the void by inducing tolerance to self-antigens which are not represented in the mosaic thymic antigen library. Tr1 cells can also mediate tolerance to persistent foreign antigens. Indeed, Tr1 cells have been identified that are specific for allergens such as Derp2 from house dust mite, Ara h1 and Ara h2 from peanuts, gliadin from gluten, bee venom, and commensal microbes (13, 29, 44, 47, 148–150). While Tr1 cells may potentially respond to a wider array of persistent antigens than FOXP3^+^ Tregs, there is no doubt that both cell types are critical in inducing and maintaining tolerance and they can cooperate to control disease through disparate mechanisms, thereby establishing tolerance when one cell type alone may be insufficient (44, 69, 72, 77, 151–153).

## Concluding remarks

As our collective understanding of Tr1 biology continues to grow, we can appreciate their role in tolerance in various disease settings as well as possible limitations such as in infectious diseases. The suppressive function of Tr1 cells has been mostly investigated in T1D, cell and organ transplantation, and IBD models while their role in other autoimmune diseases has not been extensively investigated. By continuing to push forward, we can strive to uniformly characterize human Tr1 cells to ultimately use them as novel therapies for inducing tolerance in autoimmune and autoinflammatory diseases. Further knowledge will support the development of novel Tr1-based tolerogenic therapies and allow us to apply them in a safe and efficacious manner.

## Author contributions

All authors contributed to the article and approved the submitted version.

## Acknowledgments

The authors would like to thank Jason Nideffer for critical reading and thoughtful scientific discussions during the preparation of this review.

## Conflict of interest

The authors declare that the research was conducted in the absence of any commercial or financial relationships that could be construed as a potential conflict of interest.

## Publisher’s note

All claims expressed in this article are solely those of the authors and do not necessarily represent those of their affiliated organizations, or those of the publisher, the editors and the reviewers. Any product that may be evaluated in this article, or claim that may be made by its manufacturer, is not guaranteed or endorsed by the publisher.

## Glossary

**Table:**

AhR	aryl hydrocarbon receptor
AID	autoimmune disease
APC	antigen presenting cell
ASGPR	asialoglycoprotein receptor
CD	Chron’s disease
CNS	central nervous system
DC	dendritic cell
DC-10	IL-10-producing tolerogenic dendritic cell
Dex	dexamethasone
DSS	dextran sodium sulfate
EAE	experimental autoimmune encephalitis
GAD65	glutamic acid decarboxylase 65
GI	gastrointestinal
GvHD	graft-versus-host disease
HCV	hepatitis C virus
IBD	inflammatory bowel disease
IPEX	immunodysregulation polyendocrinopathy enteropathy X-linked syndrome
LCMV	lymphocytic choriomeningitis virus
MARCO	macrophage receptor with collagenous structure
MOG	myelin oligodendrocyte glycoprotein
MS	multiple sclerosis
MSC	mesenchymal stem cell
MSCH	mouse spinal cord homogenate
NOD	non-obese diabetic
NLP	nanoliposome
NP	nanoparticle
OVA	ovalbumin
PBMC	peripheral blood mononuclear cell
RA	rheumatoid arthritis
SCID	severe combined immunodeficiency
SFU	spot-forming unit
SLE	systemic lupus erythematosus
T1D	type 1 diabetes
TCR	T cell receptor
TNBS	2, 4, 6-trinitrobenzenesulfonic acid
Tr1	type 1 regulatory T cell
Treg	regulatory T cell
UC	ulcerative colitis
VEO-IBD	very early onset inflammatory bowel disease
VitD3	vitamin D3
